# A study of a PERMA-based positive psychological intervention programme in subthreshold depressed patients with newly diagnosed breast cancer: formulation and application

**DOI:** 10.3389/fpsyg.2024.1437025

**Published:** 2024-10-15

**Authors:** Deju Wang, Bin Li, Hongmei Xin, Xiaofei Wu, Jieqiong Xia

**Affiliations:** ^1^International Nursing School, Hainan Medical University, Haikou, China; ^2^Department of Nursing Management, Hainan General Hospital, Haikou, China

**Keywords:** breast cancer, PERMA, psychology, prevention, subthreshold depression

## Abstract

**Background:**

Depression seriously affects the quality of life of breast cancer patients and even hinders treatment and recovery after diagnosis. Subthreshold depression should be worthy of attention, and the risk of subthreshold depression developing into depression increases if timely intervention is not available. However, there is limited research on interventions for subthreshold depression, especially for newly diagnosed breast cancer patients.

**Objective:**

Exploring the efficacy of a PERMA-based positive psychological intervention programme in newly diagnosed breast cancer patients with subthreshold depression.

**Methods:**

Using PERMA as a framework, we constructed the intervention programme through a literature review and expert discussion approach, and revised the programme using the Delphi method. Then we selected 84 newly diagnosed breast cancer patients for a randomised controlled trial. The control group received conventional care (primary care, specialist care, psychological care, etc.), and the observation group received a PERMA-based positive psychological intervention programme. Patient data were collected before and after the intervention, and the dataset consisted of patient responses to general information, the Depression Screening Scale (CES-D), the Hamilton Depression Inventory (HAMD-17), and the Questionnaire on Psychosocial Adjustment in Breast Cancer Patients.

**Results:**

A total of 79 patients completed the study (38 patients in the observation group and 41 patients in the control group), and before the intervention, there were no statistical differences in the comparison of general information, subthreshold depression scores, and psychosocial adaptation scores between the two groups (*p >* 0.05). After the intervention, the subthreshold depression (CES-D and HAMD-17 scores) scores of the observation group were lower than those of the control group (*p* < 0.01), and the psychosocial adaptation scores were significantly higher than those of the control group (*p* < 0.01).

**Conclusion:**

A positive psychological intervention programme using PERMA as a framework was more effective in reducing subthreshold depression levels and promoting levels of psychosocial adjustment in newly diagnosed breast cancer patients.

**Implications for practice:**

Interventions for subthreshold depression are not limited to the traditional aftercare model, but rather from a positive psychology perspective. Especially for patients newly diagnosed with breast cancer, this is a new endeavour that has important implications for them. Because, instead of focusing on their negative emotions, we help them gradually adapt to their new identity and treatment process from a positive aspect during their most difficult period, thus reducing their subthreshold depression level and preventing it from developing into a more severe depression.

## Introduction

Major Depressive Disorder (MDD) is a common mental disorder and one of the major contributors to the global burden of disease, which is characterised by persistent low mood, loss of interest, and can even lead to suicide (Peveler et al., 2002; Wu et al., 2020). Subthreshold Depression (SD) is a psychological sub-health state between health and MDD for which there is no gold standard for diagnosis (Sadek and Bona, 2000; Zhang, 1998; Yuan et al., 2020). Newly diagnosed breast cancer patients face tremendous stress on their psychological and social functioning during the diagnosis and treatment process, which can impede their subsequent treatment and recovery and seriously affect their quality of life (Hamer et al., 2017). The prevalence of SD in patients with newly diagnosed of breast cancer is 48.6% or 60.56% (Lv et al., 2017; Chen et al., 2019), and such people are 5 times more likely to develop MDD than healthy people after 1 year (Liu et al., 2015). Failure to intervene in time for SD will increase the risk of MDD (Zhang et al., 2020; Ullah et al., 2022). In clinical, between diagnosis and completion of surgery, patients go through neoadjuvant chemotherapy, endocrine therapy, targeted therapy and other treatments (Zheng and Liu, 2024), which is a long and painful process. The longitudinal survey reported (Luo et al., 2024; Charles et al., 2022; Kanani et al., 2016), during this period, patients’ subthreshold depressive symptoms or clinical depressive symptoms were associated with poorer survival, and early screening was important, and then interventions were necessary for patients with HADS-D scores >11 by screening (HAMD subthreshold depression scores range 7–17). Therefore, healthcare providers need to be more attentive to SD in this population and use proactive preventive measures in a timely manner, which is important to prevent SD from evolving into more severe MDD (Zhang, 1998). In recent years, traditional psychology has turn to positive psychology and researchers are gradually discovering its value, positive psychology not only criticises the current model of *post hoc* “treatment” advocated by negative psychology, but also promotes the idea of positive “prevention” (Li et al., 2023; Liu et al., 2016; Fang et al., 2023). In 2011 Seligman (Seligman et al., 2005) proposed the positive psychology intervention model PERMA, which summarizes the five main elements that influence well-being as Positive emotions (P), Engagement (E), Relationships (R), Meaning (M) and Achievement (A). The model uses the five elements as an implementation framework for different interventions. It aims to uncover positive emotions and create positive outcomes (Miao et al., 2013). This approach has been demonstrated in various populations to be effective in reducing patients’ anxiety and depression and promoting their psychosocial resilience (Li et al., 2024; Chen et al., 2024; Liu et al., 2024). By reviewing the literature we found that there are fewer interventions on SD in newly diagnosed breast cancer patients. One study (Wu et al., 2024) reported that they performed an intervention on patients with SD after breast cancer surgery and reduced the level of depression in the patients, but they have only focused on postoperative SD in breast cancer patients, In addition, positive psychology has rarely been studied in SD patients with newly diagnosed breast cancer. Therefore, our study aims to explore the effectiveness of a intervention programmes constructed on the basis of PERMA and to evaluate the application of this programmes in SD patients with newly diagnosed breast cancer, which is a novel attempt.

## Method

### Study design

Before the study began, a research team was assembled. (i) One graduate nursing student was primarily responsible for the design and implementation of the study and the preparation of the data analysis article; (ii) One head nurse of the breast surgery department provided assistance in the implementation of the intervention; (iii) Two psychologists were responsible for the revision and improvement of the intervention programme was modified and implemented by the specialised clinical nurses who participated in the study. The distribution and collection of the questionnaires was undertaken by one graduate and one undergraduate nursing student. Overall quality control of the study was the responsibility of the graduate student supervisors.

The study was comprised of two distinct parts. (i) The first part involved the construction of a prevention programme based on the PERMA model. (ii) The programme was conducted as a randomised controlled study. Firstly, the intervention strategy was developed and the initial draft of the protocol was formulated using PERMA theory as a framework. This was followed by two rounds of Delphi expert communication, after which the intervention protocol was revised based on expert opinions and internal discussions within the research team. Subsequently, patients were selected through a rigorous recruitment process and the intervention was implemented as a randomised controlled trial. During the study, data was collected from patients before and after the intervention. This dataset included general information about the patients as well as responses to the CES-D, HAMD-17 and Psychosocial Adaptation questionnaires (Chinese version).

### Operational definition

In study, we defined SD using the methodology of other researchers (Yuan et al., 2020; Ullah et al., 2022; Chen et al., 2024). There were two steps, firstly, the patients were assessed by using the CES-D. Secondly, these patients with CES-D scores more than16 were assessed again using the HAMD-17, and patients with scores between 7 and 17 were defined as SD (CES-D: >16, HAMD-17: 7 to 17).

### Participants and setting

1. Participants included both the experts who developed the programme and the patients who received the intervention.
2. We selected experts from various professional backgrounds, including psycho-educational professors, clinicians, and clinical nursing specialists, based on the following criteria: (I) They were engaged in clinical medical care and psychology education; (II) They were familiar with positive psychology; (III) They had more than 10 years of experience in their respective professional fields, held an intermediate professional title or above, and possessed a bachelor’s degree or higher (Table 1).
3. From April to December 2022, patients in the breast surgery department of a hospital in Hainan Province, China, who had been newly diagnosed with breast cancer and exhibited SD, were chosen as the subjects of this study.

**Table 1:** Overview of expert profiles (*n* = 12).

No.	Degree	Working years	Profession	No.	Degree	Working years	Profession
1	Bachelor’s degree	17	Nursing	7	Master’s degree	15	Medical
2	Bachelor’s degree	17	Nursing	8	Master’s degree	14	Medical
3	Master’s degree	15	Nursing	9	Master’s degree	17	Psychologist
4	Bachelor’s degree	21	Nursing	10	Master’s degree	21	Psychologist
5	Bachelor’s degree	16	Nursing	11	Master’s degree	13	Psychologist
6	Bachelor’s degree	22	Medical	12	Doctor’s degree	12	Psychologist

Inclusion criteria: (1) The first pathological diagnosis of breast cancer; (2) Depression Screening Scale (CES-D) score ≥ 16 points, and Hamilton Depression Scale (HAMD-17) score 7–17 points (Yuan et al., 2020; Ullah et al., 2022); (3) Patients receiving neoadjuvant chemotherapy and 6 cycles of chemotherapy; (4) Age 18–65 years old; (5) The expected survival time is more than 6 months (Wu et al., 2024).

Exclusion criteria: (1) have serious heart, brain, kidney organ dysfunction; (2) people with communication and visual impairment; (3) have participated in other researchers within the last 3 months; (4) do not know the disease.

Shedding criteria: (1) patients with serious life-threatening conditions; (2) those who quit midway due to irresistible factors; (3) participants participated in the intervention less than 3 times.

### Samples size

We referred to a similar study, and used the sample size calculation formula for the comparison of the means of two independent samples: $n_{1}$ = $n_{2}$ =[( $U_{a}$ + $U_{\beta }$ ) ^2^ σ ^2^]/ δ ^2^ (Chen, 2019), took the two-sided test, and the test level was set at *α* = 0.05 and *β* = 0.10, which was substituted into the formula to calculate a sample size of 72 cases for the two groups, and finally determined to be 84 cases considering the 15% sample shedding rate.

### Recruitment, randomisation, and allocation

The study was double blinded and the study statisticians were randomly assigned to a group of 42 (1:1). Study allocation was based on a computer-generated random number table. Blind allocation was carried out using opaque sealed envelopes containing the allocation information from the computerised random number tables. To determine the experimental group, the envelopes were opened by the researcher with the consent of the subjects. In order to avoid any possible response bias or contamination, the researchers conducted the final tests by personnel who had no prior contact with the subjects. In addition, the two groups of patients were placed in two different wards (Ward A and Ward C) to avoid communication between the two groups.

### Ethical considerations

The study was approved by the Medical Ethics Committee (HYLL-2024-163) before the start of the study, and the principles of benefit, respect, fairness and altruism were strictly observed in the implementation process, and patients voluntarily signed informed consent.

### Quality control

The quality control of this study was carried out throughout the whole process of intervention implementation. We use PERMA as a guiding framework, reviewed the international literature to understand the psychological characteristics of patients with first diagnosis of breast cancer, solicited the opinions of clinical staff and psychologists, controlled the quality and feasibility of the programme by experts meeting, and ensured the reliability of the programme by the Delphi method. Before the intervention, staff were uniformly trained to ensure consistency of the intervention. During data collection, without any prompting or guidance, two researchers entered the data together and a statistician analyzed the results to ensure the accuracy of the study data. In order to avoid intergroup contamination, we placed the beds of the two groups of patients in partitioned areas, with the observation group in area A and the control group in area C, to reduce the communication between the two groups of patients on the content of the intervention. In addition, a psychologist supervised the quality of the intervention throughout its implementation.

### Intervention

#### Observation group

We adopted a positive psychological intervention programme based on PERMA, the implementation of which consisted of five components: tapping and enhancing patients’ positive emotions (positive emotions, P) and enabling patients to immerse themselves in a state of engagement (engagement, E); rebuilding and maintaining interpersonal relationships (relationships, R); discovering the meaning of life (meaning, M); and realizing the self (achievement, A). The intervention was conducted in the afternoon of the first day of each chemotherapy cycle in the patient’s health education room, with 4–6 participants in each group, for 30–50 min each time, and two psychotherapists were involved in guiding the whole implementation process, as shown in Table 2.

**Table 2:** PERMA-based positive psychology intervention programme.

Times	Theme	Content	Objectives
The first neoadjuvant chemotherapy	Collection of physical and psychological data on patients	1. Introduce the purpose of this intervention to the patient, collect the patient’s disease-related information, communicate with the patient in depth, and gain trust; 2.To comprehensively understand the patient’s awareness of this illness and his/her belief in overcoming the illness; 3. To collect information about the patient’s psychological and social aspects from a positive perspective; 4. To analyze the problems affecting patients’ cognition and the factors hindering positive cognition, and to correct their misperceptions.	Establishment of a patient profile for the implementation of positive psychological interventions;
The second neoadjuvant chemotherapy	(P) Tapping and promoting underlying positive emotions	5. Pay attention to patients’ daily emotions and guide them to sort out their emotions; 6. Understand the adverse experiences of the patient during chemotherapy, eliminate or alleviate the causes of the negative effects by correcting the previous thinking patterns and unreasonable personal concepts, and reduce the negative experiences of the adverse experiences from a positive point of view through rational explanations, positive stress reduction, meditation, and other ways; 7. Describe to patients stories of positive psychology such as the “Pygmalion effect” and explain to patients the relationship between positive emotions and illness; 8. Distribute two books, “The Power of Positive Emotions” and “The Emotions of Happiness,” and the patients will read and share the books once, and exchange their experiences with everyone.	Complete a book note and share it orally at a book sharing session;
The third neoadjuvant chemotherapy	(E) Immersion to experience positive emotions	9. Select volunteers with breast cancer to share their successful experiences, so that patients can experience the positive journey of others, and perceive the benefits of others in positive emotions, so as to enhance patients’ positive beliefs and self-efficacy; 10. Instruct patients to recall the three events with the greatest sense of well-being, describe the events as thoroughly as possible, focus on the positive psychological experiences and feelings at that time, and immerse themselves in them, so as to guide patients to enter the state of “Blessed Flow” and its application in daily life; 11. Gratitude exercise, encouraging patients to express gratitude to their loved ones or family members, such as writing a thank-you note to friends and relatives, and encouraging family members to give positive feedback; 12. Urge patients to keep a diary of at least one positive experience during the treatment period and share it in the next chemotherapy cycle.	Recall and record 3 of the happiest events, detailing the positive experiences and processes at the time; Write 1 letter of appreciation; Complete at least 1 positive experience diary during each chemotherapy session;
The fourth cycle of neoadjuvant chemotherapy	(R) Help patients rebuild and maintain positive relationships	13. Promote two-way communication and exchange between patients and their families through interactive participation in small games such as grabbing stools, you than me guessing, etc., about 20~30min; 14. Encourage patients to actively communicate with other patients through group communication, triggering communication and generating links, about 40~50min, and establish a WeChat group to encourage patients to communicate with each other and share positive practice tips; 15. Promote positive responsive practice for patients and families through role-playing, to be completed at least once a week.	Each patient participates in at least 1 two-way communication interactive game;Actively speak up in WeChat groups to express positive practice tips;
The fifth neoadjuvant chemotherapy	(M) Finding the meaning of life	16. Facilitate 1 group discussion on the meaning of life and living, listing anything meaningful and guiding patients to express and share their positive views; 17. Correct negative views that associate illness with the meaning of life and guide patients to rediscover the meaning of life.	Express positive perceptions about the meaning of life and living in a group communication session;
The sixth neoadjuvant chemotherapy	(A) Setting goals to achieve self-worth	18. Assist patients to set up short-term and long-term goals in treatment and rehabilitation, work as well as life, and keep a written record of them, and actively discover the meaning of them; 19. Encourage patients to resume normal life and work and return to their families and society during the period between chemotherapy treatments if their condition permits; 20. Encourage patients to join volunteer teams to provide life guidance and psychological support to newly admitted patients.	Completion of a short-term goal of at least 1 voluntary practice per patient.

#### Control group

Provide patients with basic care, specialist care and psychological care, and timely psychological care based on SD screening results. Health education was carried out, and health education manuals were distributed to explain the precautions related to chemotherapy as well as the treatment and prevention of adverse reactions, and dietary guidance was given. At the end of each chemotherapy session, we learned about the patients’ psychological status, provided psychological counselling when necessary, helped patients adapt to the chemotherapy process, and explained the precautions to be taken for discharge and the next chemotherapy session. Weekly follow-up visits were made to answer patients’ questions and provide psychological counselling.

#### Research tools

1. General information questionnaire Including the patient’s age, education level, monthly family income, employment status, marital status, menstrual status, and chemotherapy regimen.
2. The Centre for Epidemiological Studies Depression Scale (CES-D) It is a commonly used international screening scale for MDD, and the Chinese version was cross-culturally validated by Zhang et al. (2010), which contains 20 items on a 0–3 point scale, with scores greater than 16 indicating different degrees of depression, and the higher the score, the higher the degree of depression, with a Cronbach’s *α* coefficient of 0.90.
3. Hamilton Rating Scale for Depression 17-item version (HAMD-17) It is a commonly used international depression scale, the Chinese version (Zheng et al., 1988) uses a 0–4 scale, which is routinely assessed through conversation and observation, with higher scores indicating more severe depression, and the criteria for depression assessment are that a total score of <7 is normal, 7–17 is probably depressed, 17–24 is definitely depressed, and > 24 is severely depressed, and its Cronbach’s *α* coefficient is 0.90.
4. Psychosocial Adaptation Questionnaire for Breast Cancer Patients The questionnaire was developed by Cheng (2010) and consisted of 63 items with five dimensions: anxiety/depression dimension, self-esteem and self-acceptance dimension, attitude dimension, self-control and self-efficacy dimension, and belongingness dimension. A 5-point Likert scale was used, with negative items reverse scored, and the higher the score, the higher the degree of psychosocial adjustment, as follows: <132 is low adjustment, 132–175 is moderate adjustment, and ≥ 176 is high adjustment, with a Cronbach’s α coefficient of 0.902 and a CVI >0.75.

### Data analysis

The data of this study were recorded in Excel (version 2019) by two researchers and statistically analysed using SPSS 25.0. First, descriptive statistics were calculated per skewness, histograms, Q-Q plots, and box plots to test the distribution normality and possible continuous variable outliers. Before the intervention, the basic data of the two groups of patients were analyzed, age belongs to the measurement data and conforms to the normal distribution, the two-sample mean *t*-test was used for comparison; marital status, employment, menstruation, chemotherapy regimen data belongs to the counting data, the chi-square test was used for comparison; literacy, personal income belongs to the grade of the seniority data was used for comparison using the Kruskal-Wallis H test. Before and after the intervention, the CES-D, HAMD-17, total psychosocial adjustment scores and the scores of each dimension between the two groups conformed to the normal distribution, and the two samples t-test was used. Before and after the intervention, the CES-D and HAMD-17 scores within the two patient groups conformed to a normal distribution, using a paired-sample t-test before and after itself. The threshold of significance was set at 0.05.

## Results

### Programme validity

The intervention programme constructed on the basis of PERMA had good expert consistency and content validity (Table 3).

**Table 3:** Results of the Delphi method study.

round	Cr	CV	Kendalls’ W	*χ*^2^	*p*
Round 1 (*n* = 12)	0.82	0.089 ~ 0.232	0.128	71.055	0.002
Round 2 (*n* = 12)	0.82	0.000 ~ 0.115	0.487	169.446	0.001

### Sociodemographic characteristics

During the implementation of the intervention in this study, a total of 5 cases were missed: 1 case was missed in the observation group (the patient was referred to treatment for major depression), and a total of 4 cases were missed in the control group (2 cases were automatically withdrawn, 1 case was withdrawn in the second cycle of chemotherapy after a serious adverse reaction, and 1 case was terminated). Eventually, 38 patients in the experimental group and 41 in the control group, totaling 79 patients, completed the intervention. The baseline difference between the two groups was not statistically significant (*p* > 0.05) (Table 4).

**Table 4:** Comparison of baseline data between the two groups of patients.

Variable	Observation (*n* = 38)	Control (*n* = 41)	*t* / *χ*^2^/ z	*p*
	Mean ±SD /*n*(%)			
Age, y	41.35 ± 7.15	42.86 ± 6.54	1.071^a^	0.511
Marital status			0.326^b^	0.225
Married/partnered/permanent relationship	35(89.5)	37(90.2)		
Single/divorced/widowed	3(7.9)	4(9.8)		
Education			1.159^c^	0.317
≤High school graduate	2(5.3)	4(9.8)		
Partial college/college graduate	25(65.8)	23(56.1)		
Graduate	11(28.9)	14(34.1)		
In-service or not			0.321^b^	0.185
Yes	24(63.1)	27(65.9)		
No	12(31.6)	13(31.7)		
Sick rest	2(5.3)	1(2.4)		
Actual family income per year, CNY			0.314^c^	0.674
0 ~ 2,999	4(10.5)	3(7.4)		
3,000 ~ 4,999	20(52.6)	19(46.3)		
≥5,000~	14(36.9)	19(46.3)		
Menopausal/premenopausal			0.717^b^	0.259
Menopausal	12(31.6)	11(26.8)		
Premenopausal	26(68.4)	30(73.2)		
Chemotherapy regimens			0.347^b^	0.373
AC	5(13.2)	5(12.2)		
TAC	6(15.8)	5(12.2)		
CAF	21(55.3)	24(58.5)		
CEF	6(15.7)	7(17.1)		

a *t.*

b *χ2.*

c *z.*

### Comparison of subthreshold depression scores before and after intervention

The scores of CES-D and HAMD-17 showed no statistically significant difference between the two groups before intervention (p > 0.05), while the scores of the observation group were significantly lower than those of the control group after intervention (*p* < 0.001), as shown in Table 5.

**Table 5:** Comparison of CES-D and HAMD-17 scores ( $\bar{x\;}\pm$ *s*).

Group	Total (*n*)	CES-D		*t*	*p*	HAMD-17		*t*	*p*
		Before	After			Before	After		
Observation	38	23.93 ± 6.11	10.47 ± 6.61	14.325	<0.001	13.49 ± 3.41	6.12 ± 4.37	9.646	<0.001
Control	41	22.65 ± 5.38	21.35 ± 6.46	11.680	<0.001	13.87 ± 5.68	10.69 ± 5.67	4.381	0.004
*t*		0.587	9.762			0.754	8.973		
*p*		0.641	<0.001			0.810	<0.001		

### Comparison of psychosocial adaptation scores before and after intervention

Before intervention, there was no statistically significant difference in psychosocial adjustment scores between the two groups (*p* > 0.05). After intervention, the observation group was significantly higher than the control group, and the difference between the two groups was statistically significant (*p* < 0.001) (see Table 6).

**Table 6:** Comparison of psychosocial adaptation scores ( $\bar{x\;}\pm$ *s*).

Item		Observation group (*n* = 38)	Control group (*n* = 41)	*t*	*p*
Anxiety / depression	Before	23.36 ± 2.12	23.14 ± 3.15	1.699	0.233
	After	27.68 ± 3.71	23.32 ± 2.58	2.361	<0.001
Self-esteem and self-acceptance	Before	17.56 ± 5.18	16.76 ± 6.32	3.131	0.936
	After	21.65 ± 4.21	17.69 ± 5.21	5.354	<0.001
Attitude	Before	31.74 ± 3.68	30.45 ± 5.11	3.152	1.365
	After	36.79 ± 3.33	32.68 ± 4.16	1.237	<0.001
Self-control and self-efficacy	Before	21.33 ± 6.13	22.12 ± 5.67	2.256	0.003
	After	28.75 ± 2.31	22.69 ± 3.38	4.121	<0.001
Belonging	Before	17.23 ± 7.12	19.32 ± 5.11	3.218	0.045
	After	23.86 ± 8.11	20.1 ± 6.77	2.367	<0.001
Psychosocial adjustment	Before	146.34 ± 6.87	144.67 ± 17.56	−1.682	0.716
	After	178.96 ± 18.13	146.18 ± 10.11	−2.751	<0.001

## Discussion

### Validity of PERMA-based positive psychology intervention programmes

A study reported that the positive psychological intervention programme constructed around the five elements of positive emotions, participation, relationship, meaning and achievement, using PERMA as a framework, has a certain degree of scientific and normative validity (Cheng, 2010). When constructing the programme, we selected physicians, nurses, and psychoeducational experts with intermediate or higher titles, all of whom are experienced experts in the field, and discussed all the elements of the programme cautiously and agreed on the opinions. In Delphi studies, the validity of the findings depends on the authority of the expert (Cr) and the consistency of the expert’s opinion. It is generally accepted that an expert authority coefficient Cr ≥ 0.7 implies that the results of the study are reliable. The degree of expert coordination depends on the size of the coefficient of variation (Cv) and Kendall’s coefficient of concordance (*Kendall’s W*), the value of *Kendall’s W* ranges from 0 to 1, and the larger the value is, the better the degree of expert coordination is. A Cv value of less than 0.25 means that the more consistent the expert opinion is (*p* < 0.05). The results of the study showed that, the Cr value of the two rounds of consultation is 0.82, which indicates that the degree of expert authority is high, the *Kendall’s W* of the two rounds of correspondence is 0.128 and 0.487, and the Cv value is 0.089 ~ 0.232 and 0.00 ~ 0.15, which indicates that the expert opinions converge, and we terminate the expert consultation. Therefore, the scheme we constructed has good validity. In addition, in the process of intervention implementation, the two psychoeducational experts participated in the whole process of intervention guidance and quality control, and solved the problems of the patients on time. The retention rate in the intervention group was 90.5%, with patients completing all components of each intervention and meeting the intervention goals that were settled out in the program, making the intervention program feasible.

### PERMA-based positive psychological intervention reduces SD levels in newly diagnosed breast cancer patients

Impaired body image and dysfunction experienced by breast cancer patients during treatment can exacerbate the patient’s increased psychological burden, with depression being the most common and worrisome issue, but less attention has been paid to SD (Liu et al., 2015). One study found that patients with SD were five times more likely to be depressed 1 year later than healthy individuals (Chen, 2019). Patients with first diagnosed breast cancer are in the early stages of disease treatment, and acceptance of the disease still requires a certain process of psychological adjustment, and SD should receive more attention (Zheng and Liu, 2024; Wu et al., 2024).

In this study, we used a 6-phase continuous intervention based on the PERMA positive psychology intervention programme. Firstly, we collected information on patients’ perceptions related to the disease and looked for their positive perceptions as much as possible. Secondly, by tapping into their underlying positive psychological factors and amplifying these positive emotions through guidance, such as reading two positive psychology books and sharing and exchanging them in groups in the form of book clubs. Third, patients participate in immersive experiential activities and enhance positive states. Through volunteers sharing their successful experiences (the volunteers are patients with positive emotions in the process of breast cancer recovery), patients perceive the benefits of others in positive emotions and enhance their positive experiences. For example, each patient shared three things that made them happy. Encourage patients to engage in gratitude exercises to express appreciation to family members. Record at least one positive experience during treatment and share it at the next stage. Fourth, help patients rebuild and maintain positive relationships. For example, the patient participated in some interactive games with others. Fifth, we held positive discussions to assist the patient in discovering the meaning of life. Sixth, we help patients to set long-term or short-term goals and promote their self-actualisation through the achievement of these goals. From the perspective of positive psychology, through understanding the patients’ self-perception of the disease, correcting incorrect perceptions, adopting different ways of explanation, dissection and excavation, and encouraging the patients to carry out positive guiding activities in the form of gratitude practice, writing expression, sharing and communication, the patients were gradually returned to a positive state of mind, experiencing the “input” and “blessing” of the patients. It gradually makes patients return to a positive psychological state, experience “input” and “blessing flow,” and then improves and relieves patients’ depression. The results of the study showed that the CES-D and HAMD-17 scores of patients in the observation group were significantly lower than those of the control group after the intervention, Significant differences were found within and between the two groups (*p*<0.001), indicating the effectiveness of positive psychological intervention based on PERMA in the subthreshold depressed population of first-diagnosed breast cancer, and the effect is significantly better than that of the conventional care group, which in turn proves that the effect of the positive psychology intervention model is superior to that of the traditional psychology intervention model. In China, Liu et al. (2024) and others adopted PERMA positive intervention for diabetic foot patients with similar results, through the guidance of the patients to re-establish goals and confidence, actively search for the meaning of life, and realize the self-worth of the patients with positive significance. Cheavens et al. (2006) and others implemented goal reconstruction and hope reconstruction of psychological interventions for cancer patients, similarly proved that goal adjustment has a positive effect on cancer patients.

In summary, in this study, we did not directly intervene or treat the patients’ adverse experiences and negative emotions, but rather, under the PERMA perspective, through a series of positive psychological interventions, we gradually guided the patients to discover their own positive emotions and good experiences, taught the patients to master the methods of obtaining positive emotions, actively guided the patients, prompted the patients to be more focused and fulfilled, assisted the patients in re-establishing interpersonal relationships, re-establishing life goals, and promoting self-realisation, etc., thus effectively alleviating SD in patients with first-diagnosed breast cancer.

### PERMA-based positive psychological interventions improved levels of psychosocial adaptation in newly diagnosed breast cancer patients

“Psychosocial adaptation of breast cancer patients” refers to the emotional reactions, cognitive attitudes and self-evaluation of breast cancer patients when they encounter psychological stress, as well as the degree to which their own behaviors are compatible with various types of groups, such as the family, the collective, and the society, as well as coordinated with social customs and norms, which can be evaluated from the aspects of anxiety and depression, self-esteem and self-acceptance, attitudes, self-control and self-efficacy, and sense of belonging in five dimensions (Liu et al., 2021). Therefore, the subthreshold depression status of patients is also one of the important factors affecting the level of psychosocial adjustment of patients.

Li (2023) and others found that the level of psychosocial adaptation of patients with first diagnosis of breast cancer was at a low level, while the level of psychosocial adaptation of breast cancer patients could be improved by positive cognitive intervention (Zhao, 2023). In this study, we found that the positive psychological intervention simultaneously improved the patients’ psychosocial adaptation, and the difference was statistically significant (*p*<0.001). Some studies have pointed out that suffering from cancer seriously affects patients’ life goals and pursuit of self-worth, and reasonable adjustments should be made to the original life goals for different target groups, which can help to promote patients’ psychosocial adaptability, alleviate psychological burden, and improve prognostic outcomes (Ran et al., 2024). The goal of positive psychology is to develop personal strengths and enhance positive emotions, which in turn promotes positive changes in patients (Chen et al., 2024). Therefore, rather than working to urge patients to develop positive emotions from bad experiences and experiences, this intervention approach taps into the positive qualities and underlying strengths of patients and responds positively through a series of measures (Sin and Lyubomirsky, 2009). This study utilized positive-responsive exercises, role-playing, and two-way communication with family members to help patients engage in their roles and pass through the period of adjustment to the illness, and then assisted them in re-establishing their goals and pursuits and returning to normal life and work. In addition, by encouraging patients to act as volunteers to help other patients, it helps them to realize their self-worth and gain a sense of achievement, and therefore improves the psychosocial adaptive capacity of first-diagnosed breast cancer patients, which is similar to the findings of Niu et al. (2023) and others. Therefore, the subthreshold depression intervention program constructed on the basis of the positive psychology perspective of PERMA is similarly conducive to promoting the level of psychosocial adaptation of patients.

## Limitations

This intervention study yielded relatively satisfactory preliminary results, but the limitations of this study are that the sample was selected from only one hospital, which may be under-representative, and the low baseline age of the intervention population, which leaves self-destructive and psychosocial resilience in the older population to be further investigated in the future. The absence of intentional analyses of the decontextualised sample is also a shortcoming as our study is still ongoing at this stage, which will be added in subsequent studies.

Despite the rigorous baseline treatment of the two groups in this study, the actual effect of demographic factors on depression cannot be ruled out, which is an issue that deserves ongoing attention to facilitate the provision of individualised interventions. In addition, the intervention period in this study was long, and we did not repeat the measurement of patients’ depression levels, so it is not possible to determine the impact of time effects on the results of the study, as problems with these measurement tools could lead to Barnum effects in psychological testing, which in turn could affect the results of the intervention.

## Conclusion

In conclusion, SD in newly diagnosed breast cancer patients is a clinical problem that should not be ignored, and we should actively intervene as early as possible to prevent it from developing into more serious depression, which is very necessary for patients. The positive psychology intervention programme developed in this study based on PERMA is scientific, standardised, effective and operable, which can improve the SD condition of newly diagnosed breast cancer patients and promote their psychosocial adaptability. This shows that this intervention approach from a positive psychology perspective is recommendable, and further studies will be conducted to explore its long-term effects.

## Data Availability

The raw data supporting the conclusions of this article will be made available by the authors, without undue reservation.
